# Sanjad-Sakati Syndrome Revealed by Hypocalcemic Convulsions

**DOI:** 10.7759/cureus.66429

**Published:** 2024-08-08

**Authors:** Nour El Houda Benchaib, Aziza Elouali, Anane Sara, Maria Rkain, Abdeladim Babakhouya

**Affiliations:** 1 Department of Pediatrics, Mohammed VI University Hospital, Faculty of Medicine and Pharmacy, Mohammed I University, Oujda, MAR; 2 Department of Pediatric Gastroenterology, Centre Hospitalier Universitaire (CHU) Mohammed VI, Oujda, MAR

**Keywords:** convulsion, consanguinity, facial dysmorphism, hypocalcemia, sanjad-sakati syndrome

## Abstract

Sanjad-Sakati syndrome is an autosomal recessive disorder characterized by facial dysmorphia, growth retardation, and congenital hypoparathyroidism. Epidemiologically, this syndrome is primarily observed in children of Arabian descent. However, cases have also been reported in non-Arab countries. Although its exact prevalence is uncertain, the estimated incidence in Saudi Arabia ranges from one in 40,000 to one in 600,000 live births. We report a case of Sanjad-Sakati syndrome in a female infant, born to first-degree consanguineous parents, who presented with convulsive seizures since the age of four months. Laboratory findings indicated severe hypocalcemia and elevated phosphate levels, consistent with congenital hypoparathyroidism. The treatment involved calcium and vitamin D supplementation, which led to a marked improvement in the patient’s condition. The objective of this clinical case is to highlight an uncommon cause of hypocalcemia and to describe certain clinical and endocrinological manifestations of Sanjad-Sakati syndrome, which is prevalent in the Arab population.

## Introduction

Hypocalcemia is one of the most common electrolyte disorders in children, defined by corrected serum total calcium levels below 2.12 mmol/L (8.5 mg/dL). Patients with hypocalcemia may present with a variety of symptoms and signs, as low serum calcium levels can potentially impact virtually any organ and system.

Disorders causing hypocalcemia can be divided into parathyroid hormone (PTH)-related and non-PTH-related disorders. Among the genetic disorders related to PTH, Sanjad-Sakati syndrome (SSS), also known as hypoparathyroidism-retardation-dysmorphism (HRD) syndrome, is notable. Other syndromes associated with hypoparathyroidism include the 22q11.2 deletion syndrome (DiGeorge syndrome) and hypoparathyroidism, sensory neural deafness, and renal dysplasia (HDR) syndrome. In terms of acquired disorders, postsurgical hypoparathyroidism and autoimmune polyendocrine syndrome type 1 (APS1) are common causes of hypocalcemia. These different etiologies underscore the importance of thorough evaluation to determine the underlying cause of hypocalcemia and guide appropriate therapeutic interventions [1].

SSS, also known as HRD syndrome, is an autosomal recessive condition characterized by specific oro-facial manifestations associated with prenatal and postnatal growth retardation, intellectual disability, limb anomalies, and congenital hypoparathyroidism. Patients with this syndrome often experience seizures early in life due to hypoparathyroidism [2,3]. Sanjad et al. [2] first described this syndrome in Saudi Arabia in 1988. Since then, it has been reported in the Arabian Peninsula, but its prevalence remains unknown. However, cases have also been reported in non-Arab countries [4,5].

## Case presentation

**Figure 1:**
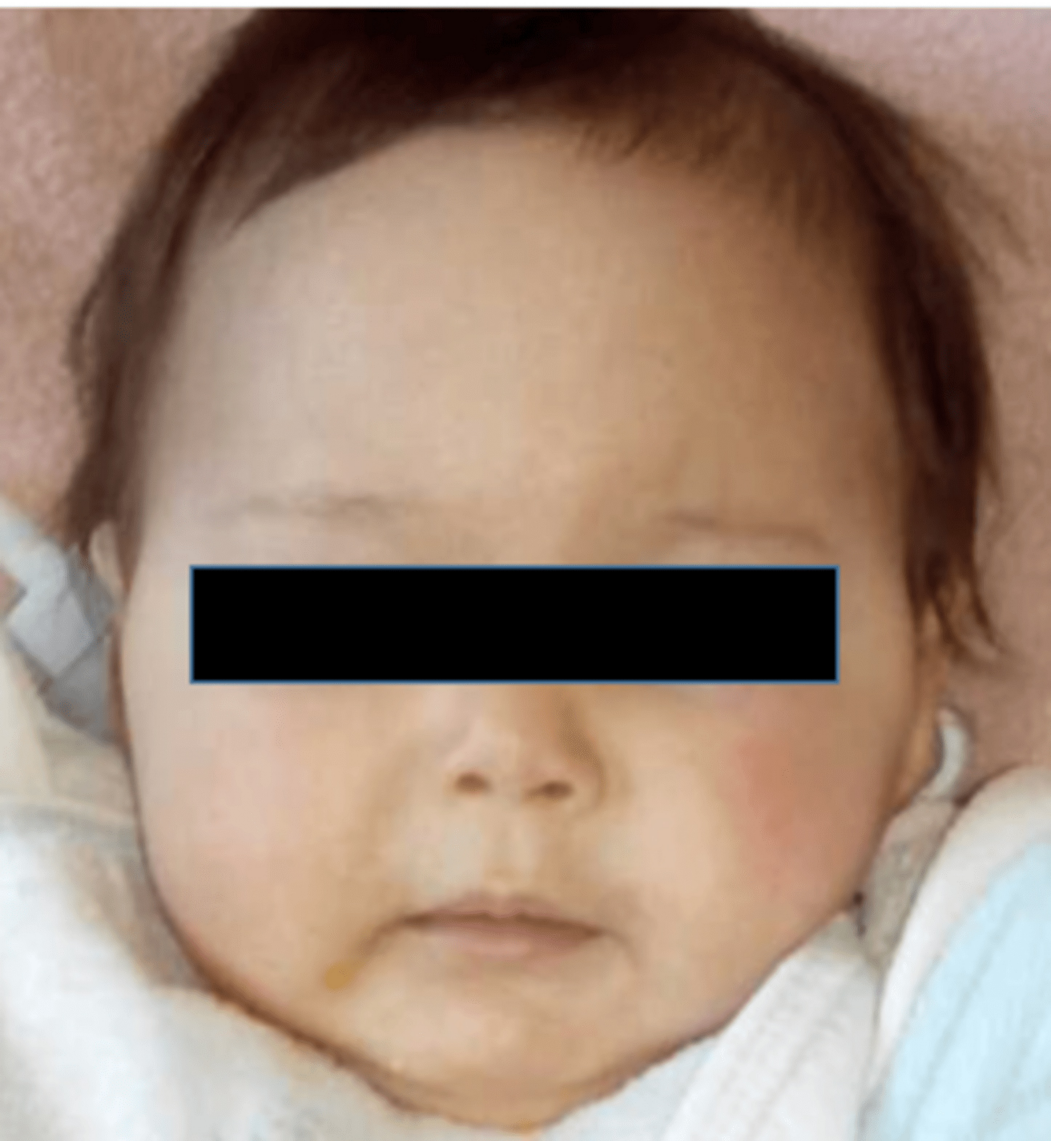
Image of our patient at the age of four months showing facial dysmorphia characterized by microcephaly, thin lips, micrognathia, small and sunken eyes, as well as dental anomalies.

This is a girl born of first-degree parental consanguinity and a well-monitored pregnancy carried to term. The delivery was medically assisted vaginally, with a birth weight of 2.7 kg and good adaptation to extrauterine life. She exhibited psychomotor developmental delay, with head control achieved at nine months, partial sitting at 14 months, and first steps at two years. She showed a delay in both height and weight growth. In her medical history, there was no record of recurrent infections. She was initially admitted at four months due to a generalized tonic-clonic seizure in an afebrile context. Facial dysmorphia was noted: microcephaly with a head circumference of 39 cm (-2 SD), a pointed nose, small and sunken eyes, low-set ears, micrognathia, and thin lips. She exhibited growth delay, weighing 4.3 kg (-3 SD) and measuring 51 cm (-4 SD) in height (Figure 1).

Biological analyses revealed hypoparathyroidism with severe hypocalcemia: serum calcium level at 39 mg/l, serum phosphate level at 75 mg/l, magnesium level at 17 mg/l, PTH level at 5.5 pg/ml, 25-hydroxy vitamin D level at 7.7 ng/ml, alkaline phosphatase (ALPn) level at 235 IU/L, and urinary calcium/creatinine ratio at 0.20 mmol/L. Additionally, we also examined the levels of other endocrine hormones with a thyroid profile, as these children can develop Hashimoto's thyroiditis. The results showed a thyroid-stimulating hormone (TSH) level of 6,22 mIU/L and a T4 level of 16 pmol/l. Unfortunately, we do not have the results for anti-thyroid antibodies. The results also showed that the insulin-like growth factor 1 (IGF-1) level was decreased. These findings suggest a more thorough evaluation of endocrine abnormalities for more comprehensive patient management (Table 1).

**Table 1:** Biological results of the patient. PTH: parathyroid hormone; TSH: thyroid-stimulating hormone; FT4: free thyroxine; IGF-1: insulin-like growth factor 1

Laboratory parameter	Values	Reference ranges
Calcium (mg/l)	39	88-108
Phosphate (mg/l)	75	23-47
Magnesium (mg/l)	17	15-22
Alkaline phosphatase (IU/l)	235	<500
PTH (pg/ml)	5.5	10-55
Albumin (g/l)	36	35-50
Creatinine (mg/l)	3.69	2.4-4.1
25-Hydroxy vitamin D (ng/ml)	7.7	20-40
TSH (mUI/l)	6.22	0.35-4.94
Free thyroxine (pmol/l)	16	12-22
IGF-1 (ug/l)	25	82-166

The patient began with the intravenous correction of hypocalcemia followed by oral supplementation with calcium carbonate and alfacalcidol.

For the radiological evaluations, we conducted a chest X-ray to investigate possible thymic agenesis, a condition associated with syndromes like DiGeorge syndrome; a transthoracic echocardiogram to identify any potential cardiac malformations; and a cervical ultrasound to assess for agenesis of the parathyroid glands and thymus. All of these tests returned normal results. Additionally, further radiological assessments were performed, including a renal ultrasound and a brain MRI. These examinations were conducted to investigate nephrocalcinosis and periventricular calcifications as potential iatrogenic complications. None of these tests revealed any significant abnormalities.

**Figure 2:**
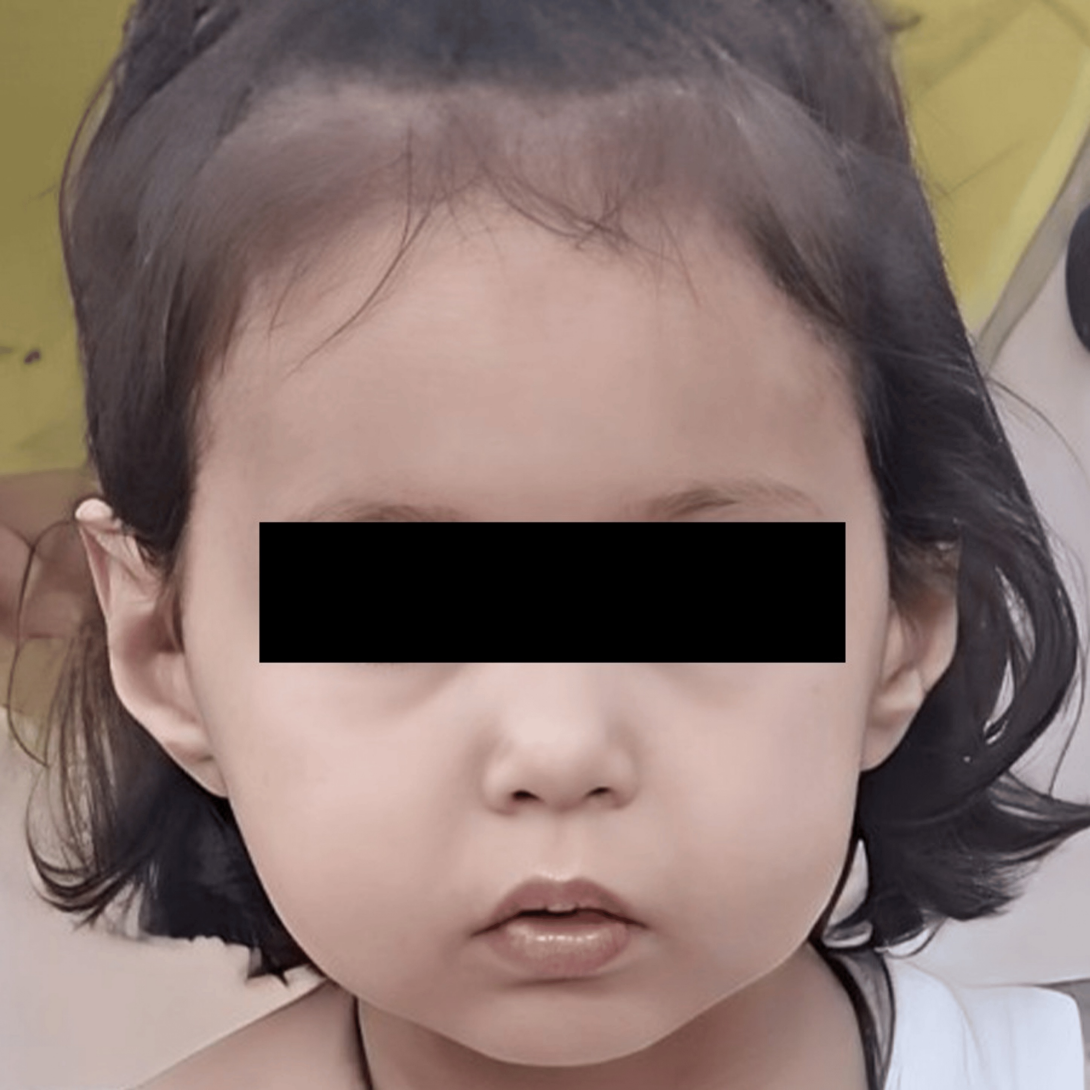
Photograph of our patient at the age of five years.

The diagnosis of SSS was confirmed based on a combination of parental consanguinity, facial dysmorphia, growth retardation in height and weight, psychomotor delay, and congenital hypoparathyroidism. Radiological examinations, including a renal ultrasound and a brain MRI to look for nephrocalcinosis and basal ganglia calcifications, did not reveal any particular abnormalities. The patient's progression has been favorable with a follow-up period of five years (Figure 2).

## Discussion

SSS, or HRD syndrome, is an autosomal recessive disease associated with mutations in the TBCE gene located on chromosome 1q42-q43. The most frequently described mutation in the TBCE gene is a 12-bp deletion in exon 3 (c.155-166del12), particularly among patients from the Middle East [3]. The TBCE gene codes for a molecular chaperone involved in the heterodimerization of alpha and beta tubulins. These microtubules are ubiquitous in all body cells, which explains the pleiotropic manifestations of the syndrome. They play a role in various intracellular processes such as cell division and motility. In the absence of this molecular chaperone, these microtubules interact with other cellular proteins that prevent them from achieving the quaternary structure necessary for their biological function [6]. In Morocco, Ratbi et al. [7] reported the first clinical and molecular description of a Moroccan patient with this syndrome and characterized the Bedouin mutation c.155-166del in the proband.

This syndrome is rare and was first described in 12 patients in Saudi Arabia by Sanjad et al. [2]. Subsequently, case series have been reported in the Arab population, including in Oman, Jordan, Qatar, Egypt, and Tunisia [8-11]. It is characterized by congenital hypoparathyroidism with hypocalcemia, hyperphosphatemia, and severely reduced PTH levels, which represents a common manifestation of the syndrome. It has been postulated that TBCE and microtubule assembly may play a crucial role in the development of the parathyroid gland, which may explain the constant association of TBCE mutations responsible for this syndrome with hypoparathyroidism [12]. A characteristic facial dysmorphism, including microcephaly, a prominent forehead, a beaked nose, sunken eyes, thin lips, micrognathia, low-set ears, and dental anomalies such as enamel hypoplasia and hypodontia, has also been reported, along with severe prenatal and postnatal growth retardation and intellectual deficits [8,9,13]. In an Israeli cohort, a 15-year follow-up of children with HRD syndrome showed inadequate growth during early childhood, with delayed and attenuated growth [14]. The authors suggested that the TBCE mutation affects microtubule assembly and chondrocyte maturation, which could be the cause of impaired longitudinal bone growth.

According to the literature, other endocrine manifestations have been reported in patients with this syndrome. Growth retardation due to growth hormone (GH) deficiency has been observed, as reported by Hafez et al. [9], who noted low serum GH levels. Similarly, Hershkovitz et al. [14] found decreased IGF-1 levels in all their patients, with a collapse in serum GH levels in one of them. These results suggest the possibility of somatotropic hypopituitarism and potential GH resistance. David et al. [13] reported in a series of 63 patients evaluating the endocrine profile of patients with HRD that IGF-1 serum concentrations were measured in 21 out of 58 patients and were low for age or undetectable in all patients. Fourteen patients underwent GH stimulation tests, and a diagnosis of GH deficiency was established in four who had abnormally low peak GH responses during two stimulation tests. For our patient with severe growth retardation, an IGF-1 concentration test was performed, which was very low, but a GH stimulation test was not conducted.

Autoimmune thyroiditis was described by Anteet et al., who studied thyroid function in patients with SSS. They emphasized the importance of systematic screening through thyroid evaluation and measurement of thyroid autoantibodies [15]. In parallel, David et al. [13] found a high prevalence of hypothyroidism, reaching 36% in patients with this syndrome. The presence of five cases of congenital hypothyroidism might suggest mild congenital thyroid hypoplasia that failed to develop properly during childhood in these individuals. This hypothesis is supported by the work of Yap and Manley [16], who demonstrated that microtubules play a crucial role in the epithelial morphogenesis of the thyroid. Moreover, SSS manifests not only as hypothyroidism and GH deficiency but also as other endocrine disorders such as adrenal glucocorticoid insufficiency and hypogonadism, as shown by David et al. [13].

Our observation represents the first case of this syndrome in the Eastern region of Morocco and aims to report a rare cause of severe hypocalcemia secondary to congenital hypoparathyroidism with specific features described in this syndrome, serving as a differential diagnosis with other syndromes such as DiGeorge syndrome, Kenny-Caffey syndrome types 1 and 2, and Barakat syndrome [17].

Management of patients with SSS presents a significant challenge for healthcare professionals. It primarily involves treating the primary hypoparathyroidism characteristic of this syndrome by correcting acute hypocalcemia when calcium levels fall below 1.80 mmol/L, regardless of clinical symptoms. This treatment typically involves intravenous calcium infusion to quickly achieve adequate blood levels to alleviate clinical and/or electrolyte symptoms. Once symptoms improve, a transition to oral treatment may be considered [18]. Additionally, it is essential to monitor high phosphate levels, as they can lead to calcifications affecting multiple body systems, particularly the basal ganglia [11]. It is worth noting that synthetic PTH treatment has not yet been approved for chronic hypoparathyroidism in children [19]. David et al. [13] treated their patients with vitamin D analogs, calcium supplements, prophylactic antibiotics (amoxicillin), and conjugated pneumococcal vaccines.

PTH treatment may be a therapeutic option for infants, replacing the physiological role of the missing hormone in maintaining normal calcium levels and limiting complications related to uncontrolled hypercalciuria. Teriparatide (PTH 1-34), the most widely available form of PTH, has been used to treat children with various etiologies of hypoparathyroidism through daily or bi-daily subcutaneous injections [20]. A case of a newborn with this syndrome, presenting with hypocalcemia refractory to conventional treatment, was treated with subcutaneous infusion of recombinant PTH (teriparatide 250 mcg/mL). Although this approach yielded satisfactory short-term results, the patient subsequently developed iatrogenic hypercalcemia.

Regarding other endocrine dysfunctions, GH deficiency generally does not respond to GH treatment in SSS patients. Hypothyroidism should be treated with levothyroxine, with treatment adjusted to the patient's current thyroid status [8].

## Conclusions

SSS is a rare autosomal recessive disorder characterized by congenital hypoparathyroidism, dysmorphic anomalies, and pre- and postnatal growth retardation. This syndrome is prevalent in the Arab population, though its prevalence is unknown. Our study, reporting the first case in the Oriental region of Morocco, highlights the importance of considering this syndrome as a cause of severe hypocalcemia, especially when associated with early seizures. These seizures, although rare, may serve as an early sign to differentiate SSS from other hypoparathyroid syndromes such as DiGeorge and Kenny-Caffey syndromes. This case underscores the need for increased awareness of this rare syndrome in clinical practice. It is crucial to continue research and develop preimplantation genetic diagnostic methods as well as carrier testing to improve the prevention and management of this disease. Early recognition and management are essential to optimize clinical outcomes, including screening for associated endocrine manifestations, avoiding iatrogenic complications, and exploring therapeutic alternatives for cases of hypocalcemia resistant to conventional treatments.

## References

[1] Pepe J, Colangelo L, Biamonte F (2020). Diagnosis and management of hypocalcemia. Endocrine.

[2] Sanjad SA, Sakati NA, Abu-Osba YK, Kaddoura R, Milner RD (1991). A new syndrome of congenital hypoparathyroidism, severe growth failure, and dysmorphic features. Arch Dis Child.

[3] Parvari R, Hershkovitz E, Kanis A, Gorodischer R, Shalitin S, Sheffield VC, Carmi R (1998). Homozygosity and linkage-disequilibrium mapping of the syndrome of congenital hypoparathyroidism, growth and mental retardation, and dysmorphism to a 1-cM interval on chromosome 1q42-43. Am J Hum Genet.

[4] Sen C, Pal S, Sengupta P, Pal A, Ganguly J, Das C, Basu D (2016). Sanjad-Sakati syndrome: beyond the Middle-East. Indian Journal of Cerebral Palsy.

[5] Arabi WA, Basheer AA, Abdullah MA (2011). Sanjad-Sakati syndrome in Sudanese children. Sudan J Paediatr.

[6] Padidela R, Kelberman D, Press M, Al-Khawari M, Hindmarsh PC, Dattani MT (2009). Mutation in the TBCE gene is associated with hypoparathyroidism-retardation-dysmorphism syndrome featuring pituitary hormone deficiencies and hypoplasia of the anterior pituitary and the corpus callosum. J Clin Endocrinol Metab.

[7] Ratbi I, Lyahyai J, Kabiri M, Banouar M, Zerkaoui M, Barkat A, Sefiania A (2015). The Bedouin mutation c.155-166del of the TBCE gene in a patient with Sanjad-Sakati syndrome of Moroccan origin. Ann Saudi Med.

[8] Bashar M, Taimur M, Amreek F, Sayeed KA, Tahir A (2020). Endocrinological manifestations of Sanjad-Sakati syndrome. Cureus.

[9] Hafez M, Anwar GM, Ibrahim A, Musa N (2017). Sanjad Sakati syndrome: case reports from Egypt. Egypt Pediatr Assoc Gaz.

[10] Kerkeni E, Sakka R, Sfar S (2015). Sanjad-Sakati syndrome in a Tunisian child. Arch Pediatr.

[11] Albaramki J, Akl K, Al-Muhtaseb A, Al-Shboul M, Mahmoud T, El-Khateeb M, Hamamy H (2012). Sanjad Sakati syndrome: a case series from Jordan. East Mediterr Health J.

[12] Parvari R, Diaz GA, Hershkovitz E (2007). Parathyroid development and the role of tubulin chaperone E. Horm Res.

[13] David O, Barash G, Agur R (2021). Multiple endocrine deficiencies are common in hypoparathyroidism-retardation-dysmorphism syndrome. J Clin Endocrinol Metab.

[14] Hershkovitz E, Rozin I, Limony Y, Golan H, Hadad N, Gorodischer R, Levy R (2007). Hypoparathyroidism, retardation, and dysmorphism syndrome: impaired early growth and increased susceptibility to severe infections due to hyposplenism and impaired polymorphonuclear cell functions. Pediatr Res.

[15] Anteet AM, Al Issa ST, Al-Ali AO, Al-Otaibi HM, Mohamed S, Babiker A, Al-Jurayyan NA (2016). Autoimmune thyroiditis associated with Sanjad-Sakati syndrome: a call for regular thyroid screening. Sudan J Paediatr.

[16] Yap AS, Manley SW (2001). Microtubule integrity is essential for apical polarization and epithelial morphogenesis in the thyroid. Cell Motil Cytoskeleton.

[17] Fukui N, Amano A, Akiyama S, Daikoku H, Wakisaka S, Morisaki I (2000). Oral findings in DiGeorge syndrome: clinical features and histologic study of primary teeth. Oral Surg Oral Med Oral Pathol Oral Radiol Endod.

[18] (2020). Pediatric hypoparathyroidism treatment & management. https://emedicine.medscape.com/article/922204-treatment.

[19] Tuli G, Buganza R, Tessaris D (2020). Teriparatide (rhPTH 1-34) treatment in children: long-term efficacy and safety data in a cohort with genetic hypoparathyroidism. Endocrine.

[20] Bali I, Al Khalifah R (2024). Recombinant PTH infusion in a child with Sanjad-Sakati syndrome refractory to conventional therapy. JCEM Case Rep.

